# Effects of *Satureja khuzistanica* essential oils in drinking water on mortality, production performance, water intake, and organ weights in broiler chickens reared under heat stress condition

**DOI:** 10.1007/s00484-015-0979-9

**Published:** 2015-03-20

**Authors:** H. Khosravinia

**Affiliations:** Department of Animal Science, Faculty of Agriculture, Lorestan University, P.B. 465, Khorramabad, 6813717133 Lorestan Iran

**Keywords:** Broiler chicken, Heat stress, *Satureja khuzistanica*, Water intake, Zootechnical performance

## Abstract

An experiment was conducted to examine the effects on mortality, production performance, water intake (WI), and organ weight of *Satureja khuzistanica* essential oil (SkEO) using 720 1-day-old Arian broiler chicks in a 42-day trial. Experimental treatments were addition of 0 (control^−^), 200, 300, 400, and 500 mg/L SkEO or 500 mg/L polysorbate 80 (control^+^) into drinking water. The birds were kept under natural ambient temperatures 4 to 6 °C above standard recommendation from days 22 to 42 of age. Addition of SkEO into drinking water at 200 and 500 mg/L decreased weight gain (*P* < 0.05) of the birds from days 29 to 35 of age with no differences in feed intake (FI) and feed conversion ratio (FCR) compared to control groups (*P* > 0.05). Supplementation of drinking water with 200, 300, 400, and 500 mg/L SkEO resulted in a 0.47, 4.40, 8.60, and 12.93 % decrease in WI, respectively, from days 1 to 42 of age. The calculated European broiler index was greater for the birds received 400 mg/L of SkEO in their drinking water compared with that of the other birds (*P* < 0.05). Pancreas percentage was increased for the birds received 200 to 500 mg/L SkEO at days 21 and 42 of age compared with that of the control^−^ birds (*P* < 0.05). The gall bladder weight was 17.56, 40.50, 12.16, and 38.73 % greater for the birds received 200, 300, 400, and 500 mg/L SkEO compared with that of the control^−^ birds, respectively. The results showed that an addition of 400 mg/L SkEO into drinking water for heat-stressed broiler chickens improves economic efficiency possibly by promoting digestion process, creating miniscule improvement in FCR and lowered mortality rate.

## Introduction

Extreme ambient temperature is probably the most prevalent environmental stressor adversely affecting welfare, health, and production economics in broiler industry (Borges et al. 2004). The major consequence of heat stress in growing birds is depressed weight gain occurring mainly due to decreased feed intake and elevated energy expenditure to reduce body temperature (Lott 1991; May and Lott 1992; Belay and Teeter 1993). Therefore, exploration of any avenue toward heat stress relief which improves feed and water intake may offer a thermoregulatory and performance advantage to heat-stressed broilers. Many studies have focused on diet modifications to alleviate hot environmental conditions for heat-stressed broilers. In most of these studies, supplementing diets or drinking water with vitamins (Mckee and Harrison 1995), electrolytes (Borges et al. 2003, 2004; Ahmed and Sarwar 2006), probiotics (Zulkifli et al. 2000), prebiotics (Riad et al. 2010), or organic acids (Goksoy et al. 2010) was the main approach suggested to minimize the impact of heat stress in broilers. Recent findings show that the use of phytogenic feed additives alleviates the harmful effect of heat stress in broiler chickens (Zeinali et al. 2011; Suderman and Solikhah 2011). Zeinali et al. (2011) reported that dietary supplementation of selenium and turmeric powder had a beneficial effect on health and plasma lipids in heat-stressed broiler chicken. Suderman and Solikhah (2011) showed that inclusion of *Pluchea indica* L. leaf meal in diet (20 g/kg) improved weight gain, feed intake, and water intake, but not feed conversion ratio, in heat-stressed broiler chicks.

*Satureja khuzistanica* Jamzad is a plant well-known for its remedial properties in traditional medicine (Zargari 1990). The aerial parts of *S. khuzistanica* collectively contain up to 3 % of an essential oil spectacularly rich in carvacrol (up to 94 %) (Khosravinia et al. 2013). Carvacrol is a phenolic, caustic, and bitter-tasting compound with good stability (Lide 1998) demonstrating antioxidant (Quiroga and Asensio 2015) and antimicrobial (Burt 2004) effects. Therefore, the *S. khuzistanica* essential oils (SkEO) exhibited antioxidant (Abdollahi et al. 2003; Radonic and Milos 2003), antiviral (Yamasaki et al. 1998), antibacterial (Azaz et al. 2002), and antifungal (Skocibusic and Bezic 2004) effects mainly in experiments conducted under standard managerial practices and normal environmental conditions.

However, experimental evidence on effects of administration of phytogenic extracts to the avian exposed to various environmental stressors such as heat-stressed birds is scarce. Therefore, this study aimed to examine the effects of SkEO on productive performance, mortality, and certain organ weights in broiler chickens exposed to seasonal extreme environmental temperatures during days 21 to 42 of age.

## Materials and methods

### Preparation of essential oil

The aerial parts of *S. khuzistanica* were manually harvested during the flowering stage of the plant in Khorraman farm, Khorramabad, Iran.^1^ The collected materials were air dried at ambient temperature under shade and hydrodistilled using a clevenger-type apparatus for 5 h, giving a yellow oil in 3 % yield. The oils were dried over anhydrous sodium sulfate and stored at 4 °C. A sample of the bulk was analyzed based on the methods used by Hadian et al. (2011) and the composition is presented in Table 1.

**Table 1:** Essential oil composition of *Satureja khuzistanica*

Compound	RI1	Composition (%)
*α*-Thujene	925	0.24 ± 0.14
*α*-Pinene	933	0.15 ± 0.05
Myrcene	981	0.26 ± 0.19
*α*-Terpinene	1013	0.24 ± 0.12
*p*-Cymene	1017	1.26 ± 0.86
Limonene	1026	0.13 ± 0.04
(Z)-*β*-ocimene	1036	0.54 ± 0.08
*γ*-Terienene	1053	0.74 ± 0.23
*trans*-Sabinene hydrate	1081	0.17 ± 0.02
Terpinen-4-ol	1163	tr
*α*-Terpinolene	1175	0.42 ± 0.45
Thymol	1266	tr
Carvacrol	1282	92.16 ± 0.46
Thymyl acetate	1329	tr
*β*-Caryophyllene	1425	0.16 ± 0.01
*α*-Humulene	1427	tr
*β*-Bisabolene	1501	tr
*trans*-*β*-Bisabolene	1522	0.10 ± 0.01

*RI1* retention indices determined relative to n-alkanes (C6–C24) on a DB-5GC column, *tr* trace (<0.05 %)

### Experimental flock

A total number of 720 1-day-old Arian broiler chicks were obtained from a commercial hatchery and housed in a concrete floor, cross-ventilated windowless shed. The chicks were randomly assigned to 36 pens (100 × 180 cm) arranged in six replicate blocks at flocking density of 12 birds per m^2^. Corn and soybean meal based diets and water were provided to the birds for ad libitum consumption throughout the experiment (Table 2). The shed was equipped with wet pad-and-fan cooling system to reduce the ambient temperature. Nonetheless, during days 21 to 42 of experiment, the average temperature during day and night hours ranged from 32 to 35 and 28 to 30 °C, respectively. Therefore, it was considered that the birds were exposed to seasonal extreme ambient temperature from day 21 of age onwards.

**Table 2:** Percentage inclusion and calculated composition of diets

Ingredient (%)	Prestarter, 1–7 days	Starter, 7–21 day	Grower, 22–35 days	Finisher, 36–42 days
Corn	53.00	55.35	57.00	61.00
Soybean meal	25.00	23.12	22.5	20.5
Fish meal	7.00	5.00	4.00	3.00
Soybean oil	4.00	4.00	4.00	5.00
Wheat	3.50	4.00	3.50	2.50
Calcium carbonate	2.00	2.00	2.00	2.00
Wheat bran	2.50	2.00	3.00	3.00
Dicalcium phosphate	2.00	2.00	2.00	2.00
DL-Methoinine	0.20	0.20	0.20	0.20
l-Lysine	0.12	0.11	0.10	0.10
Common salt	0.25	0.25	0.25	0.25
Mineral premix^a^	0.50	0.50	0.50	0.50
Vitamin permix^b^	0.75	0.65	0.55	0.50
Coccidiostat	0.10	0.10	0.10	0.00
Calculated composition				
ME, MJ/kg	12.39	12.05	12.35	12.52
Crude protein, %	22.24	21.15	20.02	17.63
Calcium, %	1.10	1.03	1.00	1.01
Available P, %	0.45	0.44	0.40	0.45
Methionine, %	0.52	0.53	0.40	0.43
Lysine, %	1.15	1.08	1.10	1.03

^a^Mineral mix supplied per kilogram diet: Mn, 55 mg; Zn, 50 mg; Fe, 80 mg; Cu, 5 mg; Se, 0.1 mg; I, 0.18 mg

^b^Vitamins mix supplied per kilogram diet: vitamin A, 18,000 IU; vitamin D_3_, 4000 IU; vitamin E, 36 mg; vitamin K_3_, 4 mg; vitamin B_12_, 0.03 mg; thiamine, 1.8 mg; riboflavin, 13.2 mg; pyridoxine, 6 mg; niacin, 60 mg; calcium pantothenate, 20 mg; folic acid, 2 mg; biotin, 0.2 mg; choline chloride, 500 mg

The effects of six experimental treatments consisting supplementation of drinking water with 0 (control^−^), 200, 300, 400, and 500 mg/L SkEO or 500 mg/L polysorbate 80 (control^+^) were examined in six replicates of 20 birds each. Polysorbate 80 used as an emulsifying agent to disperse SkEO in water at a 1:1 ratio (*v*/*v*). Mortality was recorded all through the experiment upon occurrence. Body weight gain, FI, and WI were measured weekly, and their data were used to calculate data on FCR and FI:WI ratio and a European broiler index (EBI) as (Euribrid 1994);

$$ \mathrm{E}\mathrm{B}\mathrm{I}=\left(\mathrm{viability},\ \% \times \mathrm{live}\ \mathrm{weight},\ \mathrm{kg}\right)\times 100/\left(\mathrm{age}\ \mathrm{of}\ \mathrm{slaughtering},\ \mathrm{days} \times \mathrm{feed}\ \mathrm{intake},\ \mathrm{kg}\right) $$

At day 21 of age, one male bird and at 42 days of age eight birds (4 males and 4 females) from each replicate pen were killed (without stunning) by slicing the carotid artery and jugular vein, bled for 120 s, scaled at 60 °C for 90 s, and picked using a rotary drum picker individually and eviscerated. After evisceration, the carcasses were individually weighed (CW) and the data were presented as a percentage of live weight (CY). The weight of abdominal fat (AF), liver, pancreas, duodenum, and gall bladder were also expressed proportional to carcass weight.

## Statistical analysis

The statistical model used to analyze the collected data was

$$ {Y}_{ijkl}=\mu +{\mathrm{SkEO}}_i+{S}_j+{B}_k+{\varepsilon}_{\mathrm{ijkl}}, $$

where *Y*_*ijkl*_ is the dependent variable, *μ* is the general mean, SkEO_*i*_ is the fixed effect of SkEO (*i* = 6; control^+^and 0, 200, 300, 400, 500 mg/L SkEO), *S*_*j*_ is the fixed effect of sex (*j* = 2), *B*_*k*_ is the random effect of block (*j* = 6; 1, 2, 3, 4, 5, and 6) and *ε*_*ijkl*_ is the residual error. For the variables evaluated at day 21 of age, the fixed effect of sex was omitted from the model. The data were analyzed using PROC MIXED of SAS 9.1 (2002). The LSD test was used for multiple treatment comparisons using the LSMEANS statement of SAS 9.1 (2002) with letter grouping obtained using the SAS pdmix800 macro (Saxton 1998). For the different statistical tests, significance was declared at *P* < 0.05. The REG procedure of SAS 9.1 (2002) was used to provide regression models for assessment of relationship between SkEO and water intake. Orthogonal polynomial contrasts were applied to test the linear or quadratic nature of the response in variables concerned to the graded levels of SkEO in drinking water (SAS Institute 2002).

## Results

Supplementing SkEO at the levels of 200 and 500 mg/L in drinking water did not change average daily gain (ADG) of the birds with the single exception of days 29 to 35 of age when ADG was greater in SkEO-received birds compared with the control birds (*P* < 0.05; Table 3).  Feed intake was similar among the birds (*P* > 0.05; Table 4). Feed conversion ratio tended to be lower (3.5 %) in the birds receiving 400 mg/L SkEO than in the other birds (*P* = 0.058; Table 4).

**Table 3:** Effect of essential oils of *Satureja khuzistanica* on average daily weight gain in broiler chicken up to 42 days of age

	Weight gain (g)		Essential oils of *S. khuzistanica* (mg/L)				SEM^b^	*P* value
	Control^+a^	Control^−a^	200	300	400	500		
1–7 days	16.61	16.90	16.78	16.91	16.92	16.98	0.09	0.7981
8–14 days	26.29	26.41	26.26	26.21	27.17	25.31	0.29	0.5547
15–21 days	41.31	41.72	40.19	41.52	39.40	40.90	0.32	0.1484
22–28 days	72.62	74.72	76.05	73.48	74.91	74.48	0.46	0.3590
29–35 days	81.36^b^	87.72^a^	87.48^a^	80.00^b^	85.00^ab^	80.29^b^	0.87	0.0076
36–42 days	82.43	81.43	79.60	85.62	86.07	83.00	1.25	0.6865
1–42 days	53.43	54.81	54.39	53.96	55.91	53.49	0.23	0.2456

Means within a row without a common superscript (*a*, *b*) differ significantly (*P* < 0.05)

^a^Control^+^: The birds that received drinking water supplemented with 500 mg/L polysorbate 80 throughout the trial. Control^−^: The birds that received drinking water with no additive

^b^Standard error of mean

**Table 4:** Effect of essential oils of *Satureja khuzistanica* on average daily feed intake and feed conversion ratio in broiler chicken up to day 42 of age

	Control^+a^	Essential oils of *S. khuzistanica* (mg/L)							Trend	
		0	200	300	400	500	SEM^b^	*P* value	Linear	Quadratic
Feed intake (g)										
1–7 days	19.26	19.67	19.40	19.02	19.19	19.53	0.104	0.4158	0.2345	0.2013
8–14 days	36.62	36.17	36.45	37.01	36.50	36.29	0.114	0.2258	0.6571	0.7201
15–21 days	60.01	60.95	60.03	60.71	59.86	61.46	0.252	0.3129	0.5321	0.1234
22–28 days	136.58	132.36	133.27	132.07	132.17	129.78	0.898	0.2511	0.4561	0.2340
29–35 days	183.41	175.65	176.13	175.15	175.41	175.52	1.603	0.6742	0.2913	0.4521
36–42 days	189.77	199.85	204.82	210.97	196.04	214.24	2.754	0.0672	0.3912	0.1219
1–42 days	104.27	104.11	105.08	105.82	103.18	106.14	0.466	0.4356	0.2345	0.0912
Feed conversion ratio (g feed:g gain)										
1–7 days	1.141	1.185	1.158	1.125	1.135	1.151	0.007	0.1603	0.2048	0.4193
8–14 days	1.393	1.377	1.408	1.418	1.351	1.437	0.014	0.5144	0.2834	0.2006
15–21 day	1.440	1.477	1.494	1.466	1.523	1.504	0.010	0.1280	0.2817	0.3510
22–28 days	1.829	1.824	1.754	1.798	1.765	1.745	0.013	0.1652	0.3461	0.4123
29–35 days	2.099	2.160	2.022	2.192	2.066	2.191	0.026	0.3171	0.3116	0.4218
36–42 days	2.369	2.430	2.592	2.465	2.278	2.599	0.042	0.1801	0.4760	0.1812
1–42 days	1.903	1.948	1.930	1.963	1.880	1.985	0.011	0.0581	0.1823	0.1456

^a^ *Control* ^*+*^: The birds received drinking water supplemented with 500 mg/L polysorbate 80 throughout the trial

^b^Standard error of mean

**Fig. 1:**
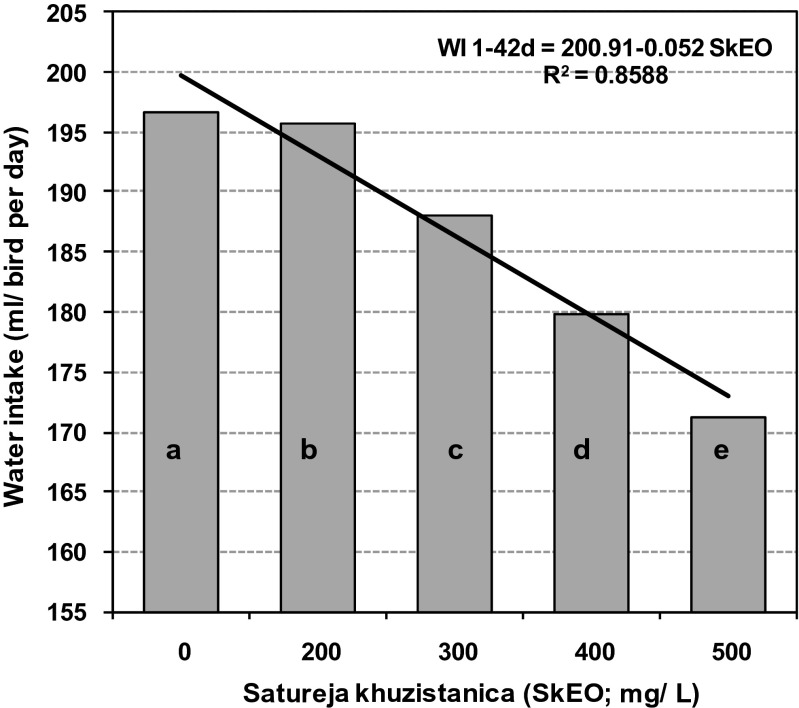
Effect of *Satureja khuzestanica* essential oils (SkEO) on water intake (WI) in broiler chicks during days 1 to 42 of age. Means without a common superscript (*a*–*e*) differ significantly (*P* < 0.05)

**Fig. 2:**
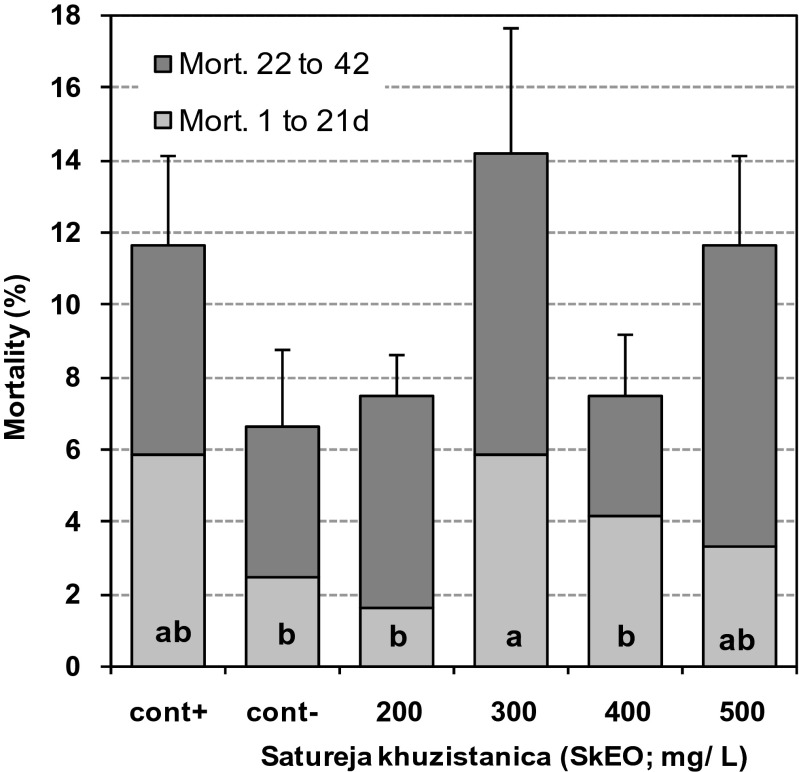
Effect of essential oils of *Satureja khuzistanica* (SkEO) on mortality of broiler chickens during 1 to 21 and 22 to 42 days of age. The *letters* inside the columns represent significant difference for mortality percent among treatments for 1 to 42 days of age. The *error bars* show standard error for mean mortality over 1 to 42 days. Means without a common superscript (*a*–*b*) differ significantly (*P* < 0.05)

Water intake was significantly decreased by addition of SkEO into drinking water in a dose-dependent manner during all weekly periods of age (*P* < 0.05; Table 5). Supplementation of drinking water with SkEO linearly decreased water intake from day 1 to 42 of age (Fig. 1). A dose-response decrease was also observed in mean FI:WI ratio for all birds received SkEO in their drinking water, but the response was not as much consistent as it was for WI (*P* < 0.01; Table 5). The mean mortality rate was not different for the treated birds compared to the control birds during days 1 to 21 and 22 to 42 of age (*P* > 0.05; Fig. 2). However, for pooled data overall periods, supplementation of drinking water with 300 and 500 mg/L SkEO increased mortality rate of the birds mainly in the second episode of the growing period when the birds were under heat stress.

**Table 5:** Effect of essential oils of *Satureja khuzistanica* on water intake and water:feed ratio in broiler chicks up to 28 days of age

	Control^+a^	Essential oils of *S. khuzistanica* (mg/L)							Trends	
		0	200	300	400	500	SEM^b^	*P*value	Linear	Quadratic
Water intake (mL/day per bird)										
1–7 days	32.86^a^	32.86^a^	30.29^b^	30.00^c^	28.57^d^	27.14^e^	0.353	0.0001	0.0234	0.5691
8–14 days	97.14^b^	102.86^a^	94.29^d^	95.71^c^	92.86^e^	91.43^f^	0.623	0.0001	0.0023	0.3491
15–21 day	162.86^c^	161.43^d^	177.14^a^	167.14^b^	167.14^b^	154.29^e^	1.172	0.0001	0.0021	0.4001
22–28 days	249.00^b^	257.43^a^	238.86^d^	242.57^c^	218.57^e^	215.71^f^	2.572	0.0001	0.0038	0.7412
29–35 days	322.07^a^	297.09^c^	305.19^b^	278.64^d^	271.43^e^	257.14^f^	3.685	0.0001	0.0231	0.4183
36–42 days	351.00^a^	328.43^c^	328.71^b^	314.14^d^	300.00^e^	281.71^f^	3.758	0.0001	0.0052	0.3426
1–42 days	202.49^a^	196.68^b^	195.75^c^	188.03^d^	179.76^e^	171.24^f^	1.810	0.0001	0.0010	0.7250
Water (mL): feed (g) ratio										
1–7 days	1.71^a^	1.67^a^	1.56^b^	1.58^b^	1.49^c^	1.39^d^	0.020	0.0001	0.0124	0.5791
8–14 days	2.65^b^	2.84^a^	2.56^cd^	2.59^c^	2.54^cd^	2.52^d^	0.020	0.0001	0.0183	0.2471
15–21 days	2.71^bc^	2.65^c^	2.95^a^	2.76^b^	2.79^b^	2.51^d^	0.025	0.0001	0.0251	0.4021
22–28 days	1.82^b^	1.95^a^	1.79^b^	1.84^b^	1.65^c^	1.67^c^	0.021	0.0001	0.0028	0.6412
29–35 days	1.76^ab^	1.69^a^	1.74^a^	1.60^bc^	1.55^cd^	1.47^d^	0.023	0.0001	0.0049	0.2103
36–42 days	1.85^a^	1.68^b^	1.62^bc^	1.50^c^	1.54^bc^	1.32^d^	0.032	0.0001	0.0131	0.4416
1–42 days	1.94^a^	1.89^ab^	1.86^b^	1.78^c^	1.75^c^	1.61^d^	0.020	0.0001	0.0217	0.6270

^a^ *Control+*: The birds that received drinking water supplemented with 500 mg/L polysorbate 80 throughout the trial. *Control* ^*−*^: The birds that received drinking water with no additive

^b^Standard error for overall mean

Means within a row without a common superscript (*a*–*f*) differ significantly (*P* < 0.05)

**Fig. 3:**
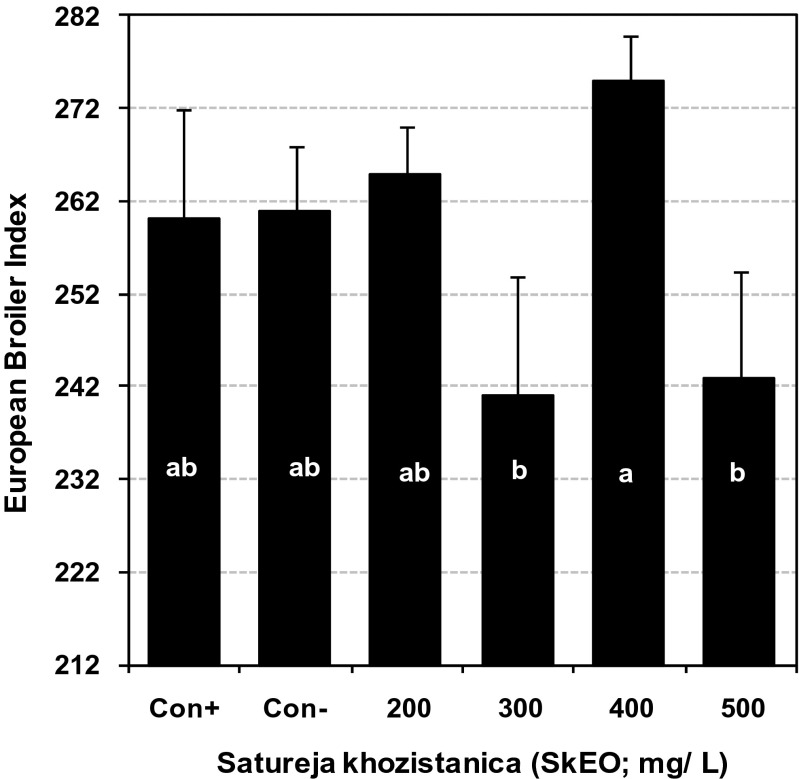
Effect of essential oils of *Satureja khuzistanica* (SkEO) on average European broiler index in broiler chicks at 42 days of age. Means without a common superscript (*a*–*b*) differ significantly (*P* < 0.05)

The calculated European broiler index was greater for the birds that received 400 mg/L of SkEO in their drinking water compared to the other groups (Fig. 3). There were no significant effects of SkEO-treated water on carcass weight, carcass yield, and abdominal fat percentage in male and female birds at day 42 of age (*P* > 0.05; Table 6). The mean duodenum weight in days 21 and 42 of age was not affected (*P* > 0.05) by SkEO-treated water (Table 7). Pancreas percentage was significantly increased (*P* < 0.05) for the birds that received 200 to 500 mg/L SkEO at 21 and 42 days of age compared with that of the control groups. Liver percentage (as the percentage of carcass weight) was not significantly different among the treated and control birds at day 42 of age (*P* < 0.05). The relative weight of gall bladder was greater for the birds that received 500 mg/L SkEO compared to the control birds at day 42 of age (*P* < 0.05; Table 7).

**Table 6:** Effect of essential oils of *Satureja khuzistanica* on carcass weight (CW; g), carcass yield (CY; %), abdominal fat-to-carcass weight ratio (AF:CW) in broiler chicks at 42 days of age

	CW	CY	AF:CW
Males			
Control^+a^	1662.6	40.54	2.442
0	1594.2	31.69	1.969
200	1651.0	38.00	2.294
300	1661.3	35.50	2.131
400	1652.2	32.00	1.940
500	1683.6	38.75	2.300
SEM^b^	14.449	1.202	0.067
*P* < *F*	0.1581	0.1859	0.2125
Trends			
Linear	0.4510	0.2871	0. 4511
Quadratic	0.2672	0.3212	0.1873
Females			
Control^+a^	1408.0	41.77	2.970
0	1454.2	36.58	2.500
200	1413.6	45.00	3.127
300	1328.4	38.08	2.866
400	1398.2	37.92	2.722
500	1409.6	36.25	2.557
SEM^b^	15.238	1.203	0.077
*P* < *F*	0.2936	0.2910	0.1581
Trends			
Linear	0.3321	0.3870	0. 6517
Quadratic	0.3642	0.3912	0.1271

^a^ *Control* ^*+*^: The birds received drinking water supplemented with 500 mg/L polysorbate 80 throughout the trial

^b^Standard error of mean

**Table 7:** Effect of essential oils of *Satureja khuzistanica* on relative weight of duodenum and pancreas (at 21 and 42 days) and relative weight of liver and bile bladder (at 42 day) in broiler chickens

	Control^+a^	Essential oils of *S. khuzistanica* (mg/L)					SEM^b^	*P* value	Trends	
		0	200	300	400	500			Linear	Quadratic
	Gram per 100 g body weight at 21 days									
Duodenum	1.412	1.689	1.628	1.625	1.483	1.507	0.045	0.3120	0.2072	0.1176
Pancreas	0.549^ab^	0.492^b^	0.579^a^	0.547^ab^	0.528^ab^	0.545^ab^	0.012	0.0192	0.3519	0.0172
	Gram per 100 g body weight at 42 days									
Duodenum	1.180	1.218	1.269	1.356	1.223	1.178	0.023	0.1927	0.3451	0.2431
Pancreas	0.332^a^	0.302^b^	0.332^a^	0.329^a^	0.332^a^	0.340^a^	0.006	0.0121	0.0345	0.3391
Liver	3.783	3.639	3.831	3.566	3.583	3.500	0.065	0.6670	0.5100	0.4310
Gall bladder	0.094^ab^	0.074^b^	0.087^ab^	0.104^a^	0.083^ab^	0.102^a^	0.004	0.0146	0.0341	0.1021

^a^ *Control* ^*+*^: The birds received drinking water supplemented with 500 mg/L polysorbate 80 throughout the trial

^b^Standard error of mean

Means within a column without a common superscript (*a*–*b*) differ significantly (*P* < 0.05)

## Discussion

In this study, SkEO exhibited no promising effect on DWG of the broiler chicken through day 28 of age when the birds were maintained under normal production practices. However, in days 29 to 35, when the birds suffered from an extreme heat stress episode, SkEO-added water decreased DWG of the birds. The decreased DWG was mainly attributed to the lowered water intake which imposed a great challenge to the birds because they needed to drink more water to overcome their disturbed homeothermic state (Lara and Rostagno 2013). In a study, supplementation of drinking water with high doses of SkEO (ranging from 500 to 2500 mg/L) adversely affected production performance of broiler chickens from days 1 to 28 of age (Khosravinia et al. 2013). When the dose of SkEO in drinking water were reduced below 500 mg/L, the birds received 500 mg/L SkEO in the first 28 days of age compensated their weight gain afterwards. These findings were inconsistent with the results of Lee et al. (2003) who reported a 2 % increase in average daily gain of broiler chicken by inclusion of 0.2 g per kg carvacrol in the diet. Addition of tymole, an isomer of carvacrol, at the same dose caused a 3 % decrease in ADG (Lee et al. 2003). The same researchers did not report water intake of the treated birds. However, the differences in the results could be explained by physiological status of the birds. In the current study, birds were not raised in standard conditions. The inconsistency in the results of current study with those reported by the other researchers could be also explained by the fact that in this experiment SkEO was added to the drinking water, while in the other studies the active components of SkEO (such as carvacrol) were mainly included in the diet. In agreement with the findings of the current study, Basmacioglu et al. (2004) reported that dietary oregano extract (a natural product rich in carvacrol) at 0.15 g/kg decreased ADG in broiler chicken by 2 % compared to the control birds. However, when the applied dose doubled (0.3 g/kg), ADG was increased as much as 2 % compared to the control group.

The results on FI and FCR in this study were not consistent. Therefore, no dose-dependent or an age-associated response could be pointed out. The unchanged FI for the treated birds was in agreement with the findings of Lee et al. (2003) but disagreed with the results reported by Basmacioglu et al. (2004). The later researchers found that dietary inclusion of 0.2 g/kg carvacrol and 0.15 g/kg oregano extract caused 2 % increase and 6 % decrease in FI of the birds compared with the relevant control groups, respectively. In contrast to the published results, in this study, adding SkEO into drinking water had no effect on FCR. These results disagree findings of Lee et al. (2003), Basmacioglu et al. (2004), and Abdel-Wareth et al. (2012) who reported decreased FCR for the birds fed with diets containing carvacrol or carvacrol-rich essential oils. The inconsistencies observed in the findings of different experiments may be attributed to the differences in management practices applied in the experimental flocks and physiological state of the birds. In the present study, SkEO decreased WI in all treated birds. Such effect has been already assigned to the flavor of water (Khosravinia et al. 2013). The bitter and pungent taste of carvacrol and possibly other principle components of SkEO caused a significant drop in water intake. Water is involved in every aspect of broiler metabolism, playing important roles in regulating body temperature, digesting food, and eliminating body wastes. At normal temperatures, poultry consume at least twice as much water as feed. Under heat stress, water intake increases (Quinteiro-Filho et al. 2012; Lara and Rostagno 2013). It seems that the caustic taste of water did not allow the treated birds to increase their WI when they were exposed to heat stress. Therefore, a dose-dependent decrease in mean FI:WI ratio was observed (Fig. 1). Unfortunately, effects of flavors on chicken performance have not been investigated in detail. Because, most researchers believe that broilers may not actually respond to the flavors as compared with mammals (Moran 1982). It was shown that dietary inclusion of carvacrol in broiler chicks reduced feed intake by modulating appetite of the birds (Lee et al. 2003). In agreement with the conclusions made by Lee et al. (2004), findings of the present study also questioned the assumption that phytogenic extracts improve the palatability of diet (Windisch et al. 2008; Costa et al. 2013). The great attention currently focused on administration of essential oils and other phytogenic products to poultry urges further investigation on response of avian species to flavors in water and feeds.

Mortality always represents a major economic loss in broiler flocks. It is usually greater in broiler flocks exposed to extreme environmental temperatures compared to those maintained in normal conditions. Water intake is an important determinant in mortality rate in heat-stressed birds (Bruno et al. 2011). Water intake increases in heat-stressed birds to maintain thermoregulatory balance (Bruno and Macari 2002), because heat stress increases water loss via exhalation as a mean to cool down body temperature (Zhang et al. 2012). In the current study, it was speculated that the decreased water intake was due to the bitter taste of carvacrol resulted in greater mortality rates in treated birds. Such a speculation became a reality as the mortality rate increased in the birds received 300 and 500 mg/L SkEO when they were exposed to extreme ambient temperature in days 21 to 42 of age.

No single production criterion can perfectly reveal the economic output of a broiler flock. Researchers have tried to pool the fractional influence of many production criteria in an index to compare performance of different flocks. The index created by Euribrid (1994) is calculated based on final body weight, FCR, and mortality. The calculated index for the treated birds with 400 mg/L of SkEO through drinking water was greater compared to other groups. The diminutive advantages of water treated with 400 mg/L SkEO in DWG, improved FCR in days 1 to 42 of age and lowered mortality rate especially during days 22 to 42 of age was reflected as an improved EBI index. These results suggest that 400 mg/L can be recommended as the dose for addition of SkEO into drinking water for broiler chickens.

In the current study, CW and CY were not affected by treatments in either male or female birds. These results were expected as carcass weight is mainly associated with pre-slaughter weight but carcass yield is mainly correlated to body composition among many other factors. However, it was anticipated that SkEO exerts a considerable impact on abdominal fat percentage. Case et al. (1995) reported that dietary inclusion of carvacrol affected fat metabolism in broiler chicken as shown by decreased abdominal fat (AF). In broiler chicken, lipids and triglycerides in particular are stored in the abdominal cavity (Saadoun and Leclercq 1987). There is a general postulation that almost all the fat built up in broiler adipose tissue including abdominal fat is synthesized in the liver or derived from the diet (Griffin et al. 1992; Hermier 1997). The results on AF disagree with the findings of Khosravinia et al. (2013) who reported that supplementation of drinking water with SkEO (500 to 2500 mg/L) significantly reduced abdominal fat in both male and female birds raised under normal conditions.

In conclusion, the present study revealed that administration of SkEO at 400 mg/L through drinking water to heat-stressed broiler chickens improves economic efficiency of broiler flocks possibly through promoting the digestion process, creating minute improvement in FCR and lowered mortality rate.

## Footnotes

1 The plant was already identified by the Department of Botany of the Research Institute of Forests and Rangelands (TARI), Tehran. A voucher specimen (No. 58416) has been deposited at the Herbarium of TARI.

## References

[CR1] Abdel-Wareth AAA, Kehraus S, Hippenstiel F, Südekum KH (2012). Effects of thyme and oregano on growth performance of broilers from 4 to 42 days of age and on microbial counts in crop, small intestine and caecum of 42-day-old broilers. Anim Feed Sci Technol.

[CR2] Abdollahi M, Salehnia A, Mortazavi SH, Ebrahimi M, Shafiee A, Fouladian F, Keshavarz F, Sorouri S, Khorasani R (2003). Antioxidant, antidiabetic, antihyperlipidemic, reproduction stimulatory properties and safety of essential oil of *Satureja khuzestanica* in rat in vivo: a toxicopharmacological study. Med Sci Monit.

[CR3] Ahmed T, Sarwar M (2006). Dietary electrolyte balance: implications in heat stressed broilers. Worlds Poult Sci J.

[CR4] Azaz D, Demirci F, Satil F, Kurkcuoglu M, Baser KH (2002). Antimicrobial activity of some Satureja essential oils. Z Naturforsch.

[CR5] Basmacioglu H, Tokusogluo O, Ergul M (2004). The effects of oregano and rosemary essential oils or alpha-tocopheryl acetate on performance and lipid oxidation of meat enriched with n-3 PUFAs in broilers. S Afr J Anim Sci.

[CR6] Belay T, Teeter RG (1993). Broiler water balance and thermobalance during thermoneutral and high ambient temperature exposure. Poult Sci.

[CR7] Borges SA, Fischer DA, Silva AV, Ariki J, Hooge DM, Cummings KR (2003). Dietary electrolyte balance for broiler chickens exposed to thermonutral or heat stress environments. Poult Sci.

[CR8] Borges SA, Fischer DA, Silva AV, Majorka A, Hooge DM, Cummings KR (2004). Physiological responses of broiler chicken to heat stress and dietary electrolyte balance (sodium plus potassium minus chloride, milliequivalants per kilogram). Poult Sci.

[CR9] Bruno LDG, Macari M, Macari M, Furlan RL, Gonzales E (2002). Ingestão de água: mecanismos regulatórios. Fisiologia aviária aplicada à frangos de corte.

[CR10] Bruno LDG, Maiorka A, Macari M, Furlan RL, Givisiez PEN (2011). Water intake behavior of broiler chickens exposed to heat stress and drinking from bell or and nipple drinkers. Rev Bras Cienc Avic.

[CR11] Burt S (2004). Essential oils: their antibacterial properties and potential applications in food—a review. Int J Food Microbiol.

[CR12] Case GL, He L, Mo H, Elson CE (1995). Induction of geranyle pyrophosphatase activity by cholesterol-suppressive isoprenoids. Lipids.

[CR13] Costa LB, Luciano FB, Miyada VS, Gois FD (2013). Herbal extracts and organic acids as natural feed additives in pig diets. S Afr J Anim Sci.

[CR14] Euribrid BV (1994). Technical information for Hybro broilers.

[CR15] Goksoy EO, Aksit M, Kirkan S (2010). The effects of organic acids and *Origanum onites* supplementations on some physical and microbial characteristics of broiler meat obtained from broilers kept under seasonal heat stress. Kafkas Univ Vet Fak Derg.

[CR16] Griffin HD, Guo K, Windsor D, Butterwith SC (1992). Adipose tissue lipogenesis and fat deposition in leaner broiler chickens. J Nutr.

[CR17] Hadian J, Mirjalili MH, Kanani MR, Salehnia A, Ganjipoor P (2011). Phytochemical and morphological characterization of *Satureja khuzistanica* Jamzad populations from Iran. Chem Biodivers.

[CR18] Hermier D (1997). Avian lipoprotein metabolism: an update on “Lipoprotein metabolism and fattening in poultry”. J Nutr.

[CR19] Khosravinia H, Ghasemei S, Rafiei AE (2013). The effect of savory (*Satureja khuzistanica*) essential oils on performance, liver and kidney functions in broiler chickens. J Anim Feed Sci.

[CR20] Lara LJ, Rostagno MH (2013). Impact of heat stress on poultry production. Animals.

[CR21] Lee KW, Everts H, Kappert HJ, Frehner M, Losa M, Beynen AC (2003). Effects of dietary essential oil components on growth performance, digestive enzymes and lipid metabolism in female broiler chickens. Br Poult Sci.

[CR22] Lee KW, Everts H, Beynen AC (2004). Essential oils in broiler nutrition. Int J Poult Sci.

[CR23] Lide DR (1998) Handbook of chemistry and physics, 87th edn. Boca Raton, FL: CRC Press, pp 3–346

[CR24] Lott BD (1991). The effect of feed intake on body temperature and water intake of male broilers during heat exposure. Poult Sci.

[CR25] May JD, Lott BD (1992). Feed and water intake patters of broilers at high environmental temperatures. Poult Sci.

[CR26] Mckee JS, Harrison PC (1995). Effects of supplemental ascorbic acid on the performance of broiler chickens exposed to multiple concurrent stressors. Poult Sci.

[CR27] Moran ETJr (1982) Comparative nutrition of fowl and swine. The gastrointestinal system. University of Guelph. pp 148–195

[CR28] Quinteiro-Filho WM, Gomes AV, Pinheiro ML, Ribeiro A, Ferraz-De-Paula V, Astolfi-Ferreira VC, Ferreira AJ, Palermo-Neto J (2012). Heat stress impairs performance and induces intestinal inflammation in broiler chickens infected with *Salmonella enteritidis*. Avian Pathol.

[CR29] Quiroga PR, Asensio CM (2015). Antioxidant effects of the monoterpenes carvacrol, thymol and sabinene hydrate on chemical and sensory stability of roasted sunflower seeds. J Sci Food Agric.

[CR30] Radonic A, Milos M (2003). Chemical composition and in vitro evaluation of antioxidant effect of free volatile compounds from Satureja montana L. Free Radic Res.

[CR31] Riad SA, Safaa HM, Mahamad FR, Siam SS, El-Minshawy HA (2010). Influence of probiotic, prebiotic and or yeast supplementation in broiler diets on the productivity, immune response and slaughter traits. J Anim Poult Prod.

[CR32] Saadoun A, Leclercq B (1987). In vivo lipogenesis of genetically lean and fat chicken: effects of nutritional state and dietary fat. J Nutr.

[CR33] SAS Institute (2002). SAS/STAT® guide for personal computers. Version 9.1 edition.

[CR34] Saxton AM (1998) A macro for converting mean separation output to letter grouping in Proc Mixed. Pages 1243-a264 in Proc. 23rd SAS User Group Intl. SAS Institute, Cary, NC

[CR35] Skocibusic M, Bezic N (2004). Phytochemical analysis and in vitro antimicrobial activity of two Satureja species essential oils. Phytother Res.

[CR36] Suderman A, Solikhah SH (2011) Performance and meat cholesterol content of broiler chickens fed Pluchea indica L. leaf meal reared under stress condition. Media Peternakan, April, 2011, hlm. 64–68

[CR37] Windisch W, Schedle K, Plitzner C, Kroismayr A (2008). Use of phytogenic products as feed additives for swine and poultry. J Anim Sci.

[CR38] Yamasaki K, Nakano M, Kawahata T, Mori H, Otake T, Ueba N, Oishi I, Inami R, Yamane M, Nakamura M, Murata H, Nakanishi T (1998). Anti-HIV-1 activity of herbs in Labiatae. Biol Pharm Bull.

[CR39] Zargari A (1990). Medicinal plants.

[CR40] Zeinali A, Kermanshahi H, Riasi A, Farhangfar H, Sarir H, Ziaie H (2011). Effects of sodium selenite and turmeric powder on thyroid hormones and plasma lipids of broiler chickens reared under heat stress condition. Glob Vet.

[CR41] Zhang ZY, Jia GQ, Zuo JJ, Zhang Y, Lei J, Ren L, Feng DY (2012). Effects of constant and cyclic heat stress on muscle metabolism and meat quality of broiler breast fillet and thigh meat. Poult Sci.

[CR42] Zulkifli IN, Abdullah NM, Azrin M, Ho YW (2000). Growth performance and immune response of two commercial broiler strains fed diets containing Lactobacillus cultures and oxytetracycline under heat stress conditions. Br Poult Sci.

